# Leaky Gut and Mycotoxins: Aflatoxin B1 Does Not Increase Gut Permeability in Broiler Chickens

**DOI:** 10.3389/fvets.2016.00010

**Published:** 2016-02-15

**Authors:** Rosario Galarza-Seeber, Juan D. Latorre, Lisa R. Bielke, Vivek A. Kuttappan, Amanda D. Wolfenden, Xochitl Hernandez-Velasco, Ruben Merino-Guzman, Jose L. Vicente, Annie Donoghue, David Cross, Billy M. Hargis, Guillermo Tellez

**Affiliations:** ^1^Department of Poultry Science, University of Arkansas, Fayetteville, AR, USA; ^2^Department of Animal Sciences, The Ohio State University, Columbus, OH, USA; ^3^Departamento de Medicina y Zootecnia de Aves, Facultad de Medicina Veterinaria y Zootecnia, Universidad Nacional Autónoma de México, Mexico City, Mexico; ^4^Pacific Vet Group-USA, Inc., Fayetteville, AR, USA; ^5^Poultry Production and Product Safety Research Unit, USDA Agricultural Research Service, Poultry Science Center, University of Arkansas, Fayetteville, AR, USA

**Keywords:** aflatoxin B1, bacterial translocation, broilers, gut leakage, performance

## Abstract

Previous studies conducted in our laboratory have demonstrated that intestinal barrier function can be adversely affected by diet ingredients or feed restriction, resulting in increased intestinal inflammation-associated permeability. Two experiments were conducted in broilers to evaluate the effect of three concentrations of Aflatoxin B1 (AFB1; 2, 1.5, or 1 ppm) on gastrointestinal leakage and liver bacterial translocation (BT). In experiment 1, 240 day-of-hatch male broilers were allocated in two groups, each group had six replicates of 20 chickens (*n* = 120/group): Control feed or feed + 2 ppm AFB1. In experiment 2, 240 day-of-hatch male broilers were allocated in three groups, each group had five replicates of 16 chickens (*n* = 80/group): Control feed; feed + 1 ppm AFB1; or feed + 1.5 ppm AFB1. In both experiments, chickens were fed starter (days 1–7) and grower diets (days 8–21) *ad libitum* and performance parameters were evaluated every week. At day 21, all chicks received an oral gavage dose of FITC-d (4.16 mg/kg) 2.5 h before collecting blood samples to evaluate gastrointestinal leakage of FITC-d. In experiment 2, a hematologic analysis was also performed. Liver sections were aseptically collected and cultured using TSA plates to determine BT. Cecal contents were collected to determine total colony-forming units per gram of Gram-negative bacteria, lactic acid bacteria (LAB), or anaerobes by plating on selective media. In experiment 2, liver, spleen, and bursa of Fabricius were removed to determine organ weight ratio, and also intestinal samples were obtained for morphometric analysis. Performance parameters, organ weight ratio, and morphometric measurements were significantly different between Control and AFB1 groups in both experiments. Gut leakage of FITC-d was not affected by the three concentrations of AFB1 evaluated (*P* > 0.05). Interestingly, a significant reduction in BT was observed in chickens that received 2 and 1 ppm AFB1. An increase (*P* < 0.05) in total aerobic bacteria, total Gram negatives, and total LAB were observed in chickens fed with 2 and 1.5 ppm of AFB1 when compared with Control and 1 ppm chickens. The integrity of gut epithelial barrier was not compromised after exposure to the mycotoxin.

## Introduction

In the winter of 1959, the British cargo ship Rosetti, unloaded a shipment of peanut meal from Brazil to England, which was utilized as a protein supplement in the diets of poultry and other domestic animals. By summer of 1960, an outbreak of an unknown disease killed several species of poultry including turkeys, ducklings, and pheasants. In all, 500 cases were reported involving the deaths of more than 100,000 turkeys. This was the first report of Turkey “X” Disease (1, 2). Exhaustive research led to the discovery of aflatoxins, secondary metabolites of *Aspergillus flavus* and *Aspergillus parasiticus*, as the etiological agents and the development of mycotoxicology (3–5). More recent studies demonstrated that aflatoxins are potent carcinogenic compounds (6–11). About 14 different types of aflatoxins are produced in nature (10, 12), but aflatoxin B1 (AFB1) produced by *A. flavus* and *A. parasiticus* is considered the most toxic (13, 14). In spite of 55 years of continuous research on aflatoxins, several areas of aflatoxicosis remain yet to be investigated. It is particularly interesting that studies on poultry aflatoxicosis have not kept pace with the research in mammals, and there still exists an incomplete description of aflatoxicosis in avian species, especially when searching for scientific publications related to the effect(s) of aflatoxins on the gastrointestinal tract (GIT).

The GIT is the first organ coming into contact with mycotoxins from the diet and should be expected to be affected by AFB1 with greater potency as compared to other organs. Nevertheless, literature regarding the effects of AFB1 on the GIT is particularly confusing. Few researchers have look at morphometric changes following dietary administration of aflatoxins in chickens, turkeys, and ducks, but results from those studies contradict each other, particularly when looking at villi high and villi to crypt ratio (10, 11, 15–20). Similarly, contradictive results arise from the effects of AFB1 on digestibility of amino acids, energy utilization, and absorption of macronutrients (18, 20–27).

Aflatoxins are absorbed very quickly into the blood from the GIT, followed by an extensive transformation into metabolites primarily in the liver (9, 28, 29). Contrary to the studies on mucosal damage and nutrient absorption caused by AFB1, there is an universal agreement that beside the carcinogenic and hepatotoxic effects on the liver, dietary aflatoxins reduce weight gain, feed intake (FI), increase feed conversion ratio (FCR), and are immunosuppressive (12, 30, 31).

Today, only a few reports could be found in databanks, in which the issue of barrier function and intestinal permeability has been reported. From recent studies by Yunus et al. (19) in broilers, it has been suggested that the absorptive surface of small intestine declines during a chronic exposure to low levels of AFB1. However, in that study, broilers compensated for the reduced absorptive surface by increasing the length of the small intestine (19). In the second study, transepithelial electrical resistance (TEER), used as an important indicator of barrier function of intestinal epithelial cells (IEC), showed that AFB1 was only moderately affect during acute exposure to the toxin (10). As far as we can tell, the only study of the effect of AFB1 on possible damage to tight junctions (TJs) was performed by Caloni et al. (32) who demonstrated that AFB1 does not affect the integrity of TJ proteins or barrier damage *in vitro*.

We have previously shown that intestinal barrier function can be adversely affected by poorly digested diets, feed restriction, or dexamethasone resulting in increased intestinal inflammation-associated permeability in poultry (33–36). The purpose of the present investigation was to evaluate the effect of three doses of aflatoxin B1 on growth, physiological parameters, and gut permeability in broiler chickens.

## Materials and Methods

### Animal Source, Diets, and Experimental Design

Two experiments were conducted several weeks apart using two hundred and forty 1-day-old male broiler chicks (Cobb-Vantress, Silom Springs, AR, USA) raised in floor pens. Unmedicated corn-soybean-based broiler starter and medicated (with coccidiostat) corn-soybean-based broiler grower diets were prepared according to the broiler’s recommendations (37). Experiments were conducted to evaluate the effect of three concentrations of AFB1 (2 ppm in experiment 1 and 1.5 or 1 ppm in experiment 2) on systemic fluorescein isothiocyanate-dextran (FITC-d; 3–5 kDa) levels and liver bacterial translocation (BT) as indicators of increased gut epithelial leakage. AFB1 was provided by Dr. George E. Rottinghaus, Veterinary Medical Diagnostic Laboratory, University of Missouri, Columbia, MO, USA. AFB1 was produced through the fermentation of rice and the aflatoxin content was measured by spectrophotometric analysis. The aflatoxin within the rice powder consisted of 74.62% AFB1, 22.38% AFG1, 2.48% AFB2, and 0.49% AFG2, based on total aflatoxin in the rice powder. Diets containing AFB1 were analyzed, and the presence of parent AF was confirmed by high-performance liquid chromatography with fluorescence detection (HPLC-FLD) method by using a Romer Derivatization Unit (Romer Labs, Inc., MO, USA). AFB1 was added to the diets and mixed thoroughly in a graded sequence to specified concentrations. The birds were given diets with or without supplemental AFB1 and water *ad libitum*. All animal handling procedures were in compliance with the Institutional Animal Care and Use Committee at the University of Arkansas. In experiment 1, broilers were allocated randomly to two groups, each group had six replicates of 20 chickens (*n* = 120/group): Control feed or feed + 2 ppm AFB1. In experiment 2, broilers were allocated randomly to three groups, each group had five replicates of 16 chickens (*n* = 80/group): Control feed; feed + 1 ppm AFB1; or feed + 1.5 ppm AFB1. In both experiments, chickens were fed starter (days 1–7) and grower diet (days 8–21) *ad libitum* until the end of the experiment at day 21. In each experiment, each pen was used as a replicate and also as an experimental unit per treatment to evaluate body weight (BW), body weight gain (BWG), FI, and FCR. These growth performance parameters were obtained every week. At the end of experiment 2, blood samples were collected from the wing vein into tubes with heparin as anticoagulant for differential cell counts. In both experiments, 21-day-old chickens received an oral gavage dose of FITC-d (4.16 mg/kg) 2.5 h before collecting blood samples to evaluate passage of FITC-d. Chickens were humanely killed by CO_2_ asphyxiation. Blood was collected from the femoral vein to obtain serum for FITC-d determination (as described below) and serum clinical chemistry (in experiment 2 only) with a Corning clinical chemistry analyzer (Chiron Corporation, San Jose, CA, USA). Liver sections (*n* = 12 chickens/treatment) were aseptically collected to determine BT, and cecal contents were collected to determine total colony-forming units per gram of Gram-negative bacteria, lactic acid bacteria (LAB), or anaerobes by plating on a selective media as described below.

### Determination of Hematological Parameters

Differential counts of blood samples collected from experiment 2 were determined using a Cell-Dyne 3500 System (Abbott Laboratories, Chicago, IL, USA) that had been standardized for differential counts of poultry blood cells. Hematologic measurements of heparin anticoagulated blood included total numbers of white blood cells (WBC), heterophils, lymphocytes, monocytes, eosinophils, and basophils. Heterophil/lymphocyte ratios (H/L), an indicator of stress in birds (38), were calculated by dividing the number of heterophils in 1 mL of peripheral blood by the number of lymphocytes. Total counts of red blood cells, hemoglobin (HGB), hematocrit (HCT)%, mean corpuscular volume (MVC), and mean corpuscular hemoglobin (MCH) were also determined. Additionally, in experiment 2, liver, spleen, and bursa of Fabricius were removed and cleaned of adherent tissues. The weight of these organs was measured and expressed as percentage of BW (organs weight/final BW) × 100.

### Serum Determination of FITC-d

Blood samples were kept at room temperature for 3 h and centrifuged (1,000 × *g* for 15 min) to separate the serum from the red blood cells. FITC-d levels of undiluted serum were measured at excitation wavelength of 485 nm and emission wavelength of 528 nm (Synergy HT, Multi-mode microplate reader, BioTek Instruments, Inc., VT, USA). Fluorescence measured was then compared to a standard curve with known FITC-d concentrations. Gut leakage for each bird was reported as microgram of FITC-d/mL of serum (35, 36).

### Bacterial Translocation

The number of birds used was based on published studies, in which similar variables were measured (34, 39). Briefly, the right half of the liver was removed from each chicken, collected in sterile bags, homogenized, weighed, and 1:4 wt/vol dilutions were made with sterile 0.9% saline. Ten-fold dilutions of each sample from each group were made in a sterile 96-well Bacti flat bottom plate, and the diluted samples were plated on tryptic soy agar plates (TSA, catalog no. 211822, Becton Dickinson, Sparks, MD, USA).

### Determination of Microbial Level in Ceca

Both ceca were aseptically removed, placed into sterile bags, and homogenized. Samples were weighed, and 1:4 wt/vol dilutions were made with sterile 0.9% saline. Ten fold dilutions of each sample from each group were made in a sterile 96-well Bacti flat bottom plate, and the diluted samples were plated on four different culture media to evaluate the total number of LAB in deMan Rogosa Sharpe (Difco™ Lactobacilli MRS Agar VWR Cat. No. 90004-084 Suwanee, GA, USA); total recovered Gram-negative bacteria in MacConkey; total anaerobes in tryptic soy agar with sodium thioglycolate plates (TSA, catalog no. 211822, Becton Dickinson, Sparks, MD, USA); and total yeast in Sabouraud Glucose Agar Base with antibiotics, tetraciclina, and 100-mg sodium benzyl penicillin (HiMedia Laboratories Pvt. Ltd., Mumbai, India).

### Histology and Morphometric Analysis of Intestine

Intestinal sections from duodenum (~1-cm section was collected from the middle of the descending duodenum), and ileum (0.5-cm section was obtained from the mid-ileum at Meckel’s diverticulum) were fixed in 10% neutral buffered formalin and embedded in paraffin, sectioned (5 μm thick), set on a glass slide, and stained with hematoxylin and eosin (H&E), and then examined by light microscopy. Photomicrographs of randomly selected fields of each intestinal sample were acquired using a microscope equipped with a Leica DFC450C camera and Leica v.3.8.Software (Leica Application Suite) and used for morphometric analysis. ImageJ 1.47v software (http://rsb.info.nih.gov/ij/) was used for the morphometric measurements of villus length, villus width, and crypt depth. Under a magnification of 2×, 10 villi per bird per section were measured, with a total of five birds per group. Villus length was measured from the top of the villus to the upper part of the lamina propria. Crypt depth was measured from the base upwards to the region of transition between the crypt and villus (40). Villus width was measured at the widest area of each villus, whereas villus:crypt ratio was determined dividing villus height into crypt depth values. Villus surface area was calculated using the formula (2π)(VW/2)(VL), where VW = villus width and VL = villus length (41).

### Statistical Analysis

All data were subjected to analysis of variance as an entirely randomized design using the General Linear Models procedure of SAS (42). Data were expressed as mean ± SE. Significant differences among the means were determined by using Duncan’s multiple-range test at *P* < 0.05.

## Results

### Performance Parameters

Body weight of chickens fed 2 ppm of AFB1 was not affected in the first week; however, BW was significantly (*P* < 0.05) reduced by 18.36 and 34.89% during the second and third weeks of age, respectively, when compared with Controls (Table 1). BW gain and FI were also affected by AFB1 consumption with a reduction of 20% for both variables during the second week and 37 and 49%, respectively, in the third week. FCR only showed a significant difference in the third week with an improvement in the AFB1 group when compared with Controls (Table 1). Administration of 1 and 1.5 ppm of AFB1 also decreased BW by 8 and 11% during the second week and 16 and 26% in the third week, respectively, compared with Controls. This reduction was proportionally similar in BWG being 10 and 13% lower for 1 and 1.5 ppm of AFB1 during the second week, and 17 and 28% for 1 and 1.5 ppm during the third week. FI was not affected by AFB1 consumption during the first 2 weeks; however, there was a reduction of 15 and 28% in fed intake in chickens that consumed 1 and 1.5 ppm of AFB1, respectively, during the last week (Table 1). FCR varied accordingly in the three diet groups during the whole experiment except the second week where Control group had a more efficient ratio compared to the AFB1 groups (Table 1). In experiment 2, the liver weight ratio was significantly increased in chickens that received 1.5 ppm when compared with Control (Table 2). However, spleen ratio was increased in both groups of chickens that received 1 or 1.5 ppm of AFB1 when compared with Controls. Bursa ratio was increased only in chickens that received 1 ppm (Table 2).

**Table 1:** **Effect of dietary administration of 2, 1.5, and 1 ppm of aflatoxin B1 on body weight (BW), body weight gain (BWG), feed intake, and feed conversion ratio at 7, 14, and 21 days in broiler chickens or experiments 1 and 2**.

	Experiment 1		Experiment 2		
	
Parameters	Control	2 ppm AFB1	Control	1 ppm AFB1	1.5 ppm AFB1
**BW (g/Broiler)**					
Day 7	144.79 ± 1.85^a^	142.05 ± 1.04^a^	136.82 ± 2.87^a^	134.92 ± 2.44^a^	133.34 ± 2.74^a^
Day 14	385.88 ± 5.02^a^	315.42 ± 5.40^b^	337.03 ± 9.38^a^	309.76 ± 2.21^b^	298.95 ± 5.03^b^
Day 21	771.55 ± 8.61^a^	502.28 ± 7.90^b^	690.45 ± 19.36^a^	581.99 ± 8.54^b^	511.03 ± 11.47^c^
**BWG (g/Broiler)**					
Days 0–7	97.83 ± 1.71^a^	95.07 ± 1.03^a^	93.24 ± 2.71^a^	90.40 ± 2.9^a^	88.62 ± 2.89^a^
Days 7–14	338.88 ± 4.85^a^	268.45 ± 5.07^b^	293.48 ± 9.20^a^	265.22 ± 2.11^b^	254.13 ± 4.74^b^
Days 14–21	724.60 ± 8.46^a^	455.30 ± 7.92^b^	646.65 ± 18.94^a^	537.47 ± 8.37^b^	466.22 ± 11.19^c^
**Feed intake (g/Broiler)**					
Days 0–7	132.1 ± 1.92^a^	127.44 ± 1.62^a^	131.35 ± 3.17^a^	128.34 ± 2.94^a^	126.42 ± 3.44^a^
Days 7–14	505.65 ± 5.86^a^	405.94 ± 6.12^b^	405.49 ± 13.15^a^	406.08 ± 6.40^a^	399.36 ± 14.80^a^
Days 14–21	966.15 ± 17.74^a^	489.09 ± 16.53^b^	790.56 ± 40.09^a^	670.32 ± 17.08^b^	570.14 ± 53.87^c^
**Feed conversion ratio**					
Days 0–7	1.35 ± 0.01^a^	1.34 ± 0.01^a^	1.41 ± 0.02^a^	1.42 ± 0.01^a^	1.43 ± 0.02^a^
Days 7–14	1.49 ± 0.02^a^	1.51 ± 0.01^a^	1.39 ± 0.06^b^	1.53 ± 0.01^a^	1.57 ± 0.03^a^
Days 14–21	1.33 ± 0.02^a^	1.08 ± 0.04^b^	1.23 ± 0.09^a^	1.25 ± 0.02^a^	1.22 ± 0.09^a^

*^a–c^Superscripts within rows indicate significant (*P* < 0.05) difference within each experiment*.

**Table 2:** **Effect of 1 and 1.5 ppm of aflatoxin B1 on body weight ratios for liver, spleen, and bursa of Fabricius in 21-day-old broiler chickens**.

Treatment	Liver ratio (%)	Spleen ratio (%)	Bursa of Fabricius ratio (%)
Control	3.24 ± 0.09^b^	0.11 ± 0.01^b^	0.15 ± 0.01^b^
1 ppm AFB1	3.60 ± 0.19^a,b^	0.16 ± 0.02^a^	0.20 ± 0.02^a^
1.5 ppm AFB1	4.23 ± 0.34^a^	0.15 ± 0.01^a^	0.18 ± 0.02^a,b^

*Experiment 2*.

*Mean ± SE from 10 chickens*.

*^a,b^Superscripts within columns indicate significant difference at *P* < 0.05*.

### Total Bacterial Counts in Cecum

In experiment 1, chicks receiving 2 ppm of AFB1 had an increase in the number of total Gram-negative bacteria and total LAB, but the total numbers of aerobes were similar between chickens that received 2 ppm of AFB1 and Control chickens (Table 3). In experiment 2, the total number of aerobic bacteria and total Gram negatives were higher in 1.5 ppm AFB1 group. Conversely, the number of total LAB was reduced in chickens fed with 1 ppm AFB1. No difference was observed in total yeast count between groups in neither of both experiments (Table 3).

**Table 3:** **Effect of 2 ppm of aflatoxin B1 (experiment 1) or 1 and 1.5 ppm of aflatoxin B1 (experiment 2) on total bacterial and yeast counts from cecum samples in broiler chickens at 21 days**.

Diet	Ceca (Log10 cfu/g of tissue)			
	Total aerobic bacteria	Total Gram-negative bacteria	Total lactic acid bacteria	Total yeast
**Experiment 1**				
Control	6.41 ± 0.19^a^	6.08 ± 0.22^b^	5.75 ± 0.21^b^	3.13 ± 0.20^a^
2 ppm AFB1	6.83 ± 0.29^a^	7.00 ± 0.21^a^	6.56 ± 0.13^a^	3.33 ± 0.07^a^
**Experiment 2**				
Control	6.98 ± 0.23^b^	6.51 ± 0.37^b^	6.91 ± 0.14^a^	2.74 ± 0.33^a^
1 ppm AFB1	7.25 ± 0.22^b^	7.04 ± 0.24^a,b^	6.33 ± 0.15^b^	3.36 ± 0.18^a^
1.5 ppm AFB1	7.82 ± 0.17^a^	7.66 ± 0.15^a^	7.22 ± 0.16^a^	2.86 ± 0.33^a^

*Data are expressed as mean ± SE from 12 chickens*.

*^a,b^Superscripts within columns indicate significant difference at *P* < 0.05*.

### Hematology

In experiment 2, a significant heterophilia with a marked lymphopenia was observed in both groups that received AFB1 (Table 4). Consequently, an increase in the heterophils-to-lymphocyte ratio was also observed in those groups when compared with Controls. No significant differences were found in the numbers of monocytes, eosinophils, or basophils (data not shown). Hemoglobin, MVC, and MCH were significantly decreased in chickens that consumed 1.5 ppm of AFB1 when compared with Controls. These values were not affected in chickens that received 1 ppm when compared with Controls (Table 4).

**Table 4:** **Effect of 1 and 1.5 ppm of aflatoxin B1 on blood parameters and serum chemistry in broiler chickens at 21 days**.

Hematological parameters	Treatments		
	Control	1 ppm AFB1	1.5 ppm AFB1
White blood cells	30.02 ± 4.57^a^	27.89 ± 2.50^a^	37.20 ± 4.23^a^
Heterophils	13.21 ± 1.38^b^	26.39 ± 2.04^a^	28.62 ± 2.70^a^
Lymphocytes	77.15 ± 2.07^a^	62.58 ± 3.31^b^	58.08 ± 2.11^b^
Heterophils lymph. ratio (HLR)	0.18 ± 0.02^b^	0.45 ± 0.05^a^	0.51 ± 0.06^a^
Red blood cells	1.81 ± 0.09^a^	1.70 ± 0.04^a^	1.68 ± 0.06^a^
Hemoglobin (HGB)	5.98 ± 0.17^a^	5.56 ± 0.18^a^	4.90 ± 0.13^b^
Hematocrit (HCT)%	44.95 ± 2.41^a^	42.07 ± 1.33^a^	39.23 ± 1.29^a^
Mean corpuscular volume (MVC)	248.1 ± 2.83^a^	247.0 ± 3.23^a^	234.4 ± 3.19^b^
Mean corpuscular hemoglobin (MCH)	33.58 ± 1.15^a^	32.63 ± 0.56^a,b^	29.42 ± 0.81^b^

*Experiment 2*.

*Mean ± SE from 10 chickens*.

*^a,b^Superscripts within rows indicate significant difference at *P* < 0.05*.

### Bacterial Translocation and FITC-d Leakage

Chickens receiving a diet with 2 ppm of AFB1 had a significant reduction in BT to the liver when compared to Control chickens (Table 5). Interestingly, there were no differences in serum levels of FITC-d levels between Control and treated chickens. On the other hand, in experiment 2, chicks fed 1.5 ppm AFB1 did not show significant differences in BT when compared with Control chickens, but no bacteria recovery was observed from livers of chickens fed with 1 ppm AFB1. Nevertheless, similar to experiment 1, no significant differences were observed in the levels of serum FITC-d between chicks that received 1 or 1.5 ppm of AFB1 and Control chickens (Table 5).

**Table 5:** **Effect of 2 ppm of aflatoxin B1 (experiment 1) or 1 and 1.5 ppm of aflatoxin B1 (experiment 2) on liver bacterial translocation and serum FITC-d levels in broiler chickens at 21 days**.

Diet	Liver bacterial translocation^c^ (log10 cfu/g of tissue)	FITC-d^c^ (**μ**g/mL of serum)
**Experiment 1**		
Control	2.77 ± 0.50^a^	0.34 ± 0.01^a^
2 ppm AFB1	1.13 ± 0.49^b^	0.39 ± 0.05^a^
**Experiment 2**		
Control	1.51 ± 0.46^a^	0.34 ± 0.02^a^
1 ppm AFB1	0.00 ± 0.00^b^	0.31 ± 0.02^a^
1.5 ppm AFB1	1.30 ± 0.47^a^	0.31 ± 0.01^a^

*^a,b^Superscripts within columns indicate significant difference at *P* < 0.05*.

*^c^Data are expressed as mean ± SE, *n* = 12 birds/treatment*.

### Morphometric Analysis

Villus length in both duodenum and ileum sections was significantly increased in a dose-related fashion in chickens that received 1 and 1.5 ppm of AFB1 when compared with Control chickens (Table 6). However, a significant reduction in duodenum crypt depth was observed in chickens that received 1 and 1.5 ppm of AFB1 when compared with Control chickens. On the other hand, similar changes in ileum crypt depth were found in chickens that received 1.5 ppm of AFB1 when compared with Control or 1 ppm chickens. Changes in duodenum villus height/crypt depth ratio were inconsistent between doses of AFB1 in this study.

**Table 6:** **Morphometric analysis of duodenum and ileum samples from broiler chickens at 21 days**.

	Duodenum			Ileum		
Parameters	Control	1 ppm AFB1	1.5 ppm AFB1	Control	1 ppm AFB1	1.5 ppm AFB1
Villus length (μm)	382.41 ± 5.03^c^	398.40 ± 2.01^b^	437.00 ± 7.50^a^	164.32 ± 3.75^c^	175.42 ± 3.13^b^	199.78 ± 3.42^a^
Villus width (μm)	45.22 ± 1.66^a^	45.83 ± 1.38^a^	47.74 ± 1.50^a^	38.93 ± 0.68^a^	37.69 ± 1.33^a^	32.57 ± 0.78^b^
Crypt depth (μm)	31.83 ± 1.03^a^	26.01 ± 0.79^b^	24.35 ± 0.15^b^	23.24 ± 0.49^b^	21.51 ± 0.69^b^	26.67 ± 0.67^a^
Villus height/crypt depth ratio	12.45 ± 0.33^c^	16.03 ± 0.48^b^	18.06 ± 0.39^a^	7.11 ± 0.12^c^	8.49 ± 0.24^a^	7.58 ± 0.10^b^
Villus surface area (mm^2^)^d^	0.054 ± 0.019^b^	0.057 ± 0.001^b^	0.066 ± 0.002^a^	0.020 ± 0.005^a^	0.021 ± 0.009^a^	0.020 ± 0.007^a^

*Experiment 2*.

*^a–c^Superscripts within rows within intestinal section indicate significant difference at *P* < 0.05*.

*^d^Surface was calculated as [2π × (villus width/2) × (villus height)] (41)*.

In the ileum, this relationship was increased in chickens that received 1 ppm, followed by chicks that received 1.5 ppm of AFB1 and Control chickens had the lower villus height/crypt depth ratio. The surface area of the duodenum was significantly higher in chicks that received 1.5 ppm of AFB1, but no changes in ileum surface area were observed between the three groups (Table 6).

## Discussion

Aflatoxins have several effects in poultry, including poor performance, liver pathology, immunosuppression, and changes in relative organ weights (30, 31, 43, 44). Our results were consistent with these previous studies demonstrating dose-related effects on reduction of BW, BWG, FI, and feed conversion as well as increase relative weights of liver, spleen, and bursa of Fabricius.

In spite of the indicated antimicrobial potential of AFB1, we found few reports regarding the effects of the toxin on gut microbial populations. Kubena et al. (44) reported a significant increase in total volatile fatty acids at 5 days of age in chickens that received 2.5 and 7.5 ppm of AFB1, suggesting changes in LAB populations (45, 46). In other studies, *Lactobacillus* spp. have been noted to change under the influence of AFB1; however, these changes did not warrant any beneficial effects of AFB1 on intestinal microbial population (47).

In the present study, AFB1 significantly increased the total number of Gram-negative bacteria in chickens fed with 2 and 1.5 ppm and numerically in chickens fed with 1 ppm, and a similar trend was observed in the total number of LAB for chickens receiving 2 and 1.5 ppm of AFB1. However, chickens that received 1 ppm showed a significant reduction of total LAB but higher total number of aerobic bacteria when compared with Control chickens. Interestingly, little information about the outcomes of AFB1 on gut microbiome is available. In one study, Kubena et al. (44) reported that 2.5 ppm of AFB1 increased the production of total volatile fatty acids in broilers, which suggest higher number of total LAB populations. In the present study, no differences were observed in total yeast counts between groups in neither of both experiments; nevertheless, we could not find any other report to compare our results. Perhaps, such inconsistent results may be a reason of the lack of publications reporting yeast evaluation. Interestingly, it has been showed that fermentation patterns of *Saccharomyces cerevisiae* also change under the influence of AFB1 (48). AFB1 has also been reported to change fermentation patterns with increase gas production, due to fermentation of other carbohydrates of LAB, that affects negatively the cheese industry (46, 47, 49). Several investigators have reported the effects that aflatoxins cause to heterophilia lymphopenia and hemolytic anemia in poultry (6, 10, 30, 50, 51). In experiment 2, a dramatic increase in the heterophils occurred while the lymphocytes were reduced. Consequently, an increase in the heterophils-to-lymphocyte ratio was also observed in those groups when compared with Control chickens. A similar response of circulating leukocytes was also found when a physiological stress was applied to chickens (38). In aflatoxicosis, the spleen is enlarged due to the hemolytic anemia (52) and some reports indicate that the spleen of chickens is almost doubled in size (53). In experiment 2, spleens of chickens that received 1 and 1.5 ppm were significantly larger when compared with Control. The elevated WBC counts caused by both doses of AFB1 also support the clinical presentation of hemolytic anemia. Additionally, hemoglobin, MVC, and MCH were significantly increased in chickens that consumed 1.5 ppm of AFB1 when compared with Control chickens, confirming that aflatoxicosis causes a hemolytic anemia in chickens as has been previously reported (30, 51, 52, 54, 55).

We have previously shown that intestinal inflammation can be induced by diet ingredients or stress, affecting intestinal permeability (33–36). As the largest barrier in the body, IEC are responsible for absorption of water and nutrients, but they also prevent the entry of antigens into the blood (56–58).

Contrary to our initial hypothesis, 2 ppm of AFB1 did not increase intestinal permeability, as was evidenced by a significant reduction in BT or similar levels of serum FITC-d when compared with Control chickens. It is possible that the inflammation of the liver that is characterized by infiltration of heterophils and other inflammatory cells may handle cleaning any bacterial leakage that arrives from the porta system to the liver. Those results encouraged us to repeat and extend the experiment with lower doses of AFB1 and by comparing the morphometric changes between Control and treated groups. Our findings from experiment 2 showed that chickens fed 1.5 ppm AFB1 showed a numerical reduction in BT when compared with Control chickens, but no bacteria were recovered from livers of chickens fed with 1 ppm AFB1. Also, similar to experiment 1, no significant differences were observed in the levels of serum FITC-d between chicks that received 1 or 1.5 ppm of AFB1 and Control chickens.

Increased intestinal leakage is also associated with BT in the portal circulation (59, 60). Likewise, FITC-d is a bulky molecule (3–5 kDa) which is not observed under normal conditions. Nevertheless, if TJs between epithelial cells are altered, FITC-d can be detected in serum, indicating damage to the TJs following FITC-d gavage administration (61). It has been reported that AFB1 does not destroy TJs (32), it has only minor effects on the gut-associated lymphoid tissue (GALT) (62), confirming that AFB1 does not induce inflammation in the GIT.

Literature reports on the effects of AFB1 on histology of GIT are limited and not conclusive (10, 11, 18, 27, 63). However, it is important to mention that the few studies that have evaluated the effect of AFB1 on intestinal histology are reports using different concentrations of AFB1, different avian species, different ages, as well as time of AFB1 administration. Interpretation of our morphometric results was also inconclusive. Nevertheless, the GIT is highlighted as a dynamic organ that is able to adapt to a chronic AFB1 as has been demonstrated by several scientists (18, 20–23, 26, 27). In summary, the results of the present study suggest that AFB1 does not increase gut leakage as is evidenced by the lack of increase permeability of FITC-d in the serum. On the other hand, further studies are needed to clarify the BT and morphometric results with AFB1.

## Author Contributions

RS: contributions to conception and design, acquisition of data, and analysis and interpretation of data; JL: contributions to conception and design, acquisition of data, and/or analysis and interpretation of data; LB: contributions to conception and design, and/or analysis and interpretation of data; VK: contributions to conception and design and acquisition of data; AW: acquisition of data; XV: final approval of the version to be submitted, drafting the article, or revising it critically for important intellectual content; RG: acquisition of data; JV: acquisition of data; AD: final approval of the version to be submitted; DC: acquisition of data; BH: final approval of the version to be submitted; and GT: contributions to conception and design, acquisition of data, and analysis and interpretation of data.

## Conflict of Interest Statement

The authors declare that the research was conducted in the absence of any commercial or financial relationships that could be construed as a potential conflict of interest.
